# Association of maternal serum uric acid levels with LBW/SGA: a large retrospective cohort study

**DOI:** 10.1186/s12884-024-06261-0

**Published:** 2024-02-23

**Authors:** Yaoxi Xiong, Panchan Zheng, Chao Chen, Chengrong He, Xingyu Yang, Weiwei Cheng

**Affiliations:** 1 International Peace Maternity and Child Health Hospital, School of Medicine, Shanghai Jiao Tong University, Shanghai, 200030 China; 2 Shanghai Key Laboratory of Embryo Original Disease, Shanghai, 200030 China

**Keywords:** Uric acid (UA), Low birth weight (LBW), Small for gestational age (SGA), Retrospective cohort study

## Abstract

**Background:**

Elevated maternal serum uric acid (UA) levels were associated with adverse perinatal outcomes. This study aimed to examine the association between UA and the risk of low birth weight (LBW) / small for gestational age (SGA).

**Methods:**

A cohort study of women delivered in Shanghai maternity hospital was included between 2017 and 2021. Electronic medical records were utilized to extract information and antenatal care records. The cut-off value of UA was 360 μmol/L. The outcome was LBW/SGA, with LBW defined as birth weight below 2500 g and SGA indicating birth weight below the 10th percentile of average weight for gestational age. The assessment of SGA was based on the Chinese standard curve for birth weight at various gestational ages. Univariate, multivariate logistic regression models, restricted cubic spline were used in this study, with adjustments made for confounding factors.

**Results:**

Sixty-nine thousand six hundred seventy-four live births and singleton pregnancies were included. The ratio of LBW/SGA was 3.3%/9%. Maternal UA levels were significantly negatively correlated with birth weight. High UA levels were associated with high risk of LBW/SGA, especially in third trimester. In BMI** < **25 group, the risk of LBW increased to 2.35-fold (95%CI, 1.66–3.31) in hyperuricemic group (UA** > **360 μmol/L). The SGA risk was 1.66-fold (95%CI, 1.37–2.00). Gestational hypertension (GH) with hyperuricemica increased the risk of LBW (aOR = 4.00, 95%CI, 2.01–7.93) and SGA (aOR = 2.63, 95%CI, 1.83–3.78). Preeclampsia (PE) with hyperuricemia increased the risk of LBW (aOR = 1.38, 95%CI, 0.63–3.03) and SGA (aOR = 1.81, 95%CI, 1.18–2.78). In delivery gestational week (DGW) ≥ 37 group, if UA > 360 μmol/L, the incidence of LBW increased to 2.46-fold (95%CI, 1.62, 3.73) and the incidence of SGA increased to 1.52-fold (95%CI, 1.24, 1.87). In DGW < 37 group, if UA > 360 μmol/L, the incidence of LBW increased to 2.70-fold (95%CI, 1.92, 3.80) and the incidence of SGA increased to 2.13-fold(95%CI, 1.50, 3.02).

**Conclusions:**

The study found an inverse correlation between UA levels and birth weight. High UA levels were associated with increased risk of LBW/SGA, particularly in third trimester. GH or PE complicated by hyperuricemia were found to have significantly higher risk of developing LBW/SGA. This relationship also existed in pregnant women with BMI** < **25.

## Background

Uric acid (UA) is an end product of purine metabolism pathway and is cleared in kidney. And UA levels are not routinely assessed during antenatal care in other countries. Elevated UA levels were reported associated with hypertension, obesity, cardiovascular disease, and insulin resistance. [1–3] The UA could pass the placenta through simple diffusion. [4, 5] If the UA levels in the maternal bloodstream are too high, it would enter the placenta, causing maternal oxidative stress, vascular endothelial excitation of placenta and upregulation of inflammatory response. [6, 7] Ultimately it could affect the development of the fetus by causing placental dysfunction.

Low birth weight (LBW) and Small for gestational age (SGA) are important indicators for assessing fetal development, which are associated with neonatal mortality and morbidity, and even other diseases in adulthood, including infectious diseases, growth retardation, neurodevelopmental deficits, insulin resistance, obesity, and hypertension. [8–10] Approximately 200,000 babies worldwide are born with LBW and 230,000 with SGA each year, posing a significant economic burden to society [11].

Previous research primarily examined the impact of hyperuricemia on birth weight in the context of hypertension, leaving the relationship between hyperuricemia and birth weight in individuals with normal blood pressure unexplored. [12–14] Furthermore, these studies predominantly relied on data from European or American populations. To our knowledge, the association between the risk of UA levels and LBW/SGA has not been investigated in a large sample in the Asian region. [15–18] Rothenbacher et al. found that maternal UA was inversely associated with a reduced risk of SGA. [15] However, in the Mendelian randomization study by Decena et al., they found that maternal UA levels were unlikely to be an important factor in the offspring's birth weight. [18] Consequently, this study was undertaken to evaluate the influence of hyperuricemia on the risk of LBW/SGA in a large sample of Chinese pregnant women. The data encompassed prenatal examination records obtained from a hospital over a span of five years. Additionally, UA levels across the entire duration of pregnancy were examined, the association between UA levels and risk of LBW/SGA for the different trimesters was be compared thereby conducting a more comprehensive investigation compared to prior studies that solely focused on UA levels during a single trimester.

## Method

### Study design and population

The study was conducted at the International Peace Maternal and Child Health Hospital of Shanghai Jiao Tong University School of Medicine. About 74,640 pregnant women who were registered at the hospital's maternity clinic between January, 2017 and December, 2021. Subsequently, pregnant women with the following conditions were excluded from the analysis: (1) Comorbid cancer, kidney disease, autoimmune diseases and other diseases that adversely affect UA levesl during pregnancy; (2) Twin and multiple pregnancies; (3) Drugs taken during pregnancy that have an effect on UA excretion; (4) Miscarriage, stillbirth or neonatal malformation; (6) Maternal age was less than 18; (7) The clinical data was not complete (no neonatal birth weight, UA levels during pregnancy were missing, and variables in Table 1 were missing more than 50%). In order to avoid selection bias, strict diagnostic criteria and exclusion criteria were established. Two researchers separately retrieved discharge diagnoses from the hospital's medical record system, and different person simultaneously re-diagnosed according to existing diagnostic criteria, and one resolved any discrepancies between the two. In short, we reviewed the medical records of the pregnant women who came to our hospital during the 5-year period and those who had complete medical records, regular prenatal checkups, and underwent UA measurements during pregnancy. The pregnant women who met the inclusion criteria were divided into hyperuricemic group (UA > 360 μmol/L) and control group (UA ≤ 360 μmol/L). [19] In this retrospective cohort study, Strengthening of Reporting of Epidemiological Observational Studies (STROBE) guidelines were followed.

### Data collection, measurement

Medical records were extracted from routine antenatal visits by obstetricians-gynecologists and nurses. Laboratory test results were obtained from the hospital's lab database. A sample of fasting venous blood was collected when pregnant women received antenatal care during pregnancy. Serum was obtained by centrifuge the whole blood sample. The Beckman Coulter AU5800 automatic biochemical analyzer in the hospital's clinical laboratory detected patient serum samples. All inspection operations were carried out in strict accordance with the relevant operating specifications. Each participant underwent a UA levels assessment in the first, second, and third trimesters.

### Outcome definitions

Primary outcomes were LBW and SGA. LBW was defined as birth weight less than 2500 g. According to the reported birth weight curves of newborns of different gestational ages in China, SGA refers to birth weight below the 10th percentile of average weight for gestational age. [20] The curve was established by establishing a nationwide neonatal collaborative network in China to collect obstetric delivery data from 63 hospitals in 23 provinces, municipalities and autonomous regions in 7 regions of the country. This curve is the standard curve of birth weight of newborns in China.

### Statistical analysis

The statistical analysis was conducted by R software (version 4.2.2). Descriptive statistics were used to describe the characters of mothers and their neonates in different categories of UA levels. Parametric and nonparametric tests were used to determine the statistical significance of the differences in different UA levels. A restrictive cubic spline curve was used to fit the dose-response relationship between UA levels and birth weight. To investigate the effect of continuous variable UA levels on birth weight, UA levels were quartered to explore changes in the risk of LBW/SGA as UA levels increased. Based on the *P*-value and our prior knowledge, the variables of *P* < 0.05 in Table 1 were considered as adjusting variables and included in the variables of the multivariate analysis model/logistic regression analysis. By adjusting for confounding factors, univariate and multivariate logistic regression models were used to analyze the relationship between UA levels and birth weight. In addition, to compare the risk of LBW/SGA between hyperuricemic group (UA > 360 μmol/L) and control group (UA ≤ 360 μmol/L), a post hoc analysis was performed. To investigate the effect of continuous variable UA levels on birth weight, its levels were quartered to explore changes in the risk of LBW/SGA as UA levels increased. The association between UA levels and risk of LBW/SGA for the different trimesters was be compared by restricted cubic spline and logistic regression models. In addition, the relationship between UA levels variation and the risk of LBW/SGA were studied. Finally, pooled analyses were also used to investigate the effect of BMI, GH, PE, DGW with hyperuricemia (UA** > **360 μmol/L) on the risk of developing LBW/SGA. The effect of *P*** < **0.05 was considered statistically significant.

## Results

### Clinical characteristics of the participants

**Fig. 1:**
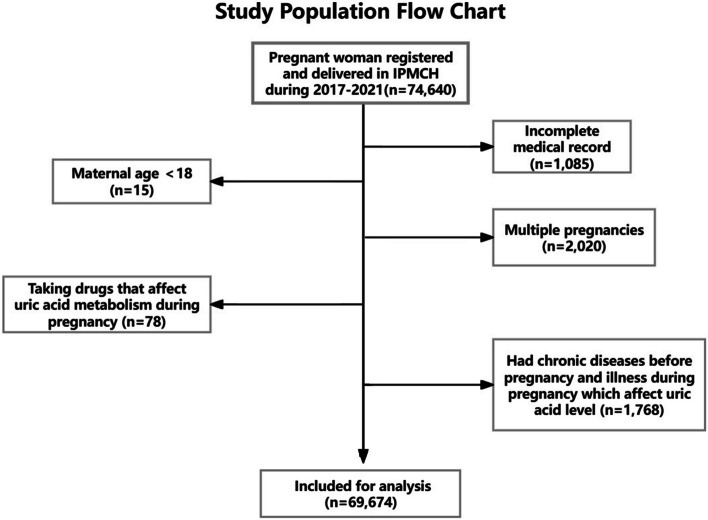
Study population flow chart

The participant registration flowchart was shown in Fig. 1. 69,674 mothers and their singletons were included for the study. The demographic characteristics of the study population were presented in Table 1. The average age of the participants was 31.3 ± 4.0 years. The proportion of nulliparous women was 69.8% (48,644) and 30.2% (21,030) of multiparous women. 76.8% (941) of pregnant women in the hyperuricemic group (UA** > **360 μmol/L) were nulliparous women. The proportion of DGW** < **37 in this cohort was 5.4% (3796), and the proportion of DGW** < **37 in the hyperuricemic group (UA** > **360 μmol/L) was 17.3% (212). The mean UA levels in the total population was 245.65 ± 47.36 μmol/L, and the mean UA levels of the hyperuricemic group (UA** > **360 μmol/L) was 392.71 ± 33.82 μmol/L. In addition, compared to the control group, the hyperuricemic group (UA** > **360 μmol/L) were more likely to have elevated BMI, abnormal blood lipid level, and a higher proportion of cesarean sections. From the perspective of conception method, pregnant women who conceive through assisted reproduction technology were more likely to have high UA levels. The prevalence of GH, PE, Anaemia, and GDM were approximately 11.7% (8185), 3.5% (2472), 1.5% (1069), and 16.2% (10,919), respectively. The proportions of LBW, macrosomia, SGA, and LGA in this cohort were 3.3% (2283), 5.2% (3632), 9.0% (6283), and 6.3% (4363), respectively. In the hyperuricemic group (UA** > **360 μmol/L), there was also a higher risk of GH, PE, Anaemia, and GDM, macrosomia. LBW and SGA were more common in people with high UA levels than in normal and low UA levels individuals.

**Table 1:** Basic characteristics of study population

	Total (*n* = 69,674)	Group		*P*-value
		UA < 360 μmol/L (*n* = 68,449)	UA ≥ 360 μmol/L (*n* = 1225)	
Age, years	31.3 ± 4.0	31.3 ± 4.0	31.3 ± 4.4	0.588
Education				< 0.001
Undergraduate and beyond	48,576(69.7)	47,851(69.9)	725(59.2)	
Below undergraduate	21,098(30.3)	20,598(30.1)	500(40.8)	
Family income, thousand				0.008
< 100	2062(3.0)	2019(2.9)	43(3.5)	
100–200	6729(9.7)	6582(9.6)	147(12.0)	
200–300	10,032(14.4)	9840(14.4)	192(15.7)	
> 300	10,217(14.7)	10,059(14.7)	158(12.9)	
Missing	40,634(58.3)	39,949(58.4)	685(55.9)	
ART				< 0.001
No	7858(11.3)	7605(11.1)	253(20.7)	
Yes	61,816(88.7)	60,844(88.9)	972(79.3)	
Smoking (Before pregnancy)				0.016
No	69,267(99.4)	68,056(99.4)	1211(98.9)	
Yes	407(0.6)	393(0.6)	14(1.1)	
BMI (Before pregnancy), kg/m^2^				< 0.001
< 18.5	9000(12.9)	8904(13.0)	96(7.8)	
18.5–24.9	51,826(74.4)	51,081(74.6)	745(60.8)	
25–29.9	5911(8.5)	5672(8.3)	239(19.5)	
> 30	814(1.2)	756(1.1)	58(4.7)	
Missing	2123(3.0)	2036(3.0)	87(7.1)	
Parity				< 0.001
nulliparous	48,644(69.8)	47,703(69.7)	941(76.8)	
Multiparous	21,030(30.2)	20,746(30.3)	284(23.2)	
SBP, mm Hg	119.97 ± 18.77	119.87 ± 18.81	125.62 ± 15.48	< 0.001
DBP, mm Hg	75.00 ± 9.42	74.93 ± 9.37	79.16 ± 11.15	< 0.001
UA, μmol/L	245.65 ± 47.36	243.02 ± 43.23	392.71 ± 33.82	< 0.001
CHOL, mmol/L				< 0.001
< 6.22	62,222(89.3)	61,177(89.4)	1045(85.3)	
≥ 6.22	7452(10.7)	7272(10.6)	180(14.7)	
TG, mmol/L				< 0.001
< 2. 32	48,033(68.9)	47,400(69.2)	633(51.7)	
≥ 2. 32	21,641(31.1)	21,049(30.8)	592(48.3)	
GH				< 0.001
No	38,634(55.4)	38,179(55.8)	455(37.1)	
Yes	8185(11.7)	7914(11.6)	271(22.1)	
Missing	22,855(32.8)	22,356(32.7)	499(40.7)	
PE				< 0.001
No	41,576(59.7)	40,999(59.9)	577(47.1)	
Yes	2472(3.5)	2328(3.4)	144(11.8)	
Missing	25,626(36.8)	25,122(36.7)	504(41.1)	
Anemia				0.041
No	68,605(98.5)	67,408(98.5)	1197(97.7)	
Yes	1069(1.5)	1041(1.5)	28(2.3)	
GDM				< 0.001
No	56,590(83.8)	55,786(84.0)	804(71.8)	
Yes	10,919(16.2)	10,603(16.0)	316(28.2)	
Delivery method				< 0.001
Caesarean	31,195(44.8)	30,454(44.5)	741(60.5)	
forceps	3208(4.6)	3161(4.6)	47(3.8)	
vaginal	35,271(50.6)	34,834(50.9)	437(35.7)	
Birthweight, g				< 0.001
< 1000	52(0.1)	40(0.1)	12(1.0)	
1000–1500	256(0.4)	217(0.3)	39(3.2)	
< 2500	1975(2.8)	1845(2.7)	130(10.6)	
2500–4000	63,759(91.5)	62,791(91.7)	968(79.0)	
≥ 4000	3632(5.2)	3556(5.2)	76(6.2)	
Birthweight				< 0.001
SGA	6283(9.0)	6076(8.9)	207(16.9)	
AGA	59,028(84.7)	58,094(84.9)	934(76.2)	
LGA	4363(6.3)	4279(6.3)	84(6.9)	
DGW, weeks				< 0.001
< 37	3796(5.4)	3584(5.2)	212(17.3)	
≥ 37	65,878(94.6)	64,865(94.8)	1013(82.7)	

*Abbreviations:ART* assisted reproductive technology, *BMI* body mass index (calculated as weight in kilograms divided by height in meters squared), *SBP* systolic blood pressure, *DBP* diastolic blood pressure, *UA* uric acid, *CHOL* cholesterol, *TG* triglyceride, *GH* gestational hypertension, *PE* preeclampsia, *GDM* gestational diabetes mellitus, *SGA* small for gestational age, *AGA* appropriate for gestational age, *LGA* large for gestational age, DGW delivery gestational week

### Relationship between UA levels and birth weight

**Fig. 2:**
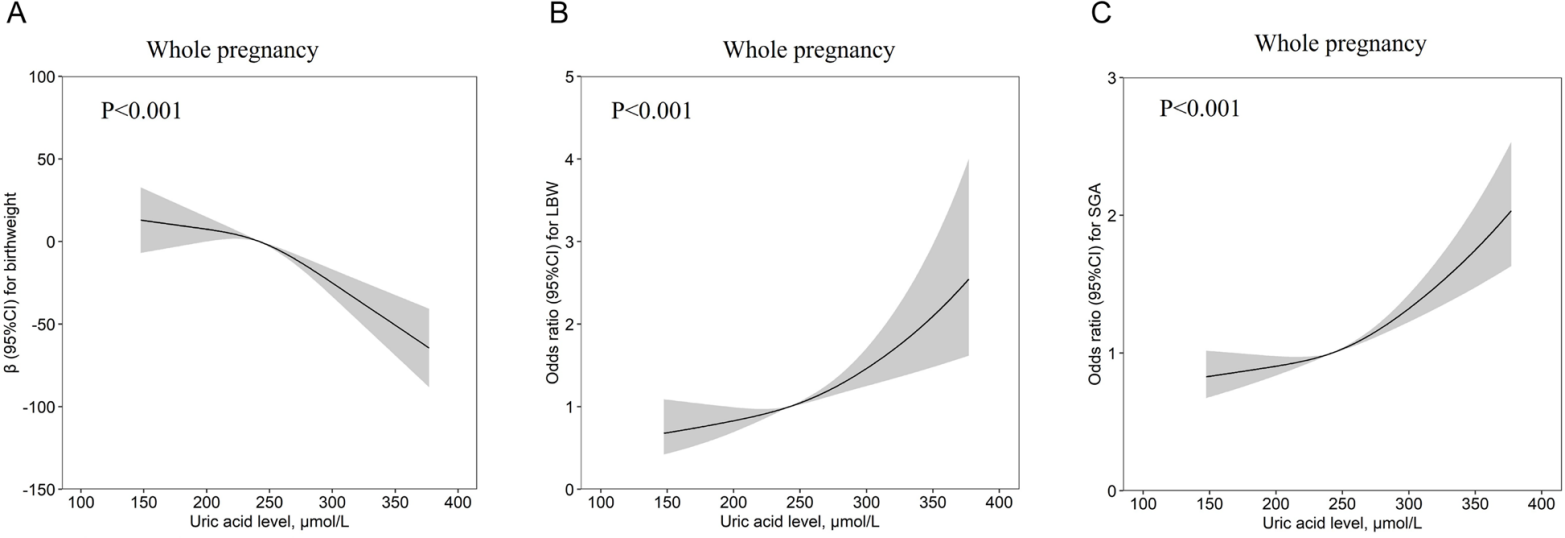
Association between Maternal UA levels and Birth Weight during the whole pregnancy. **A** The association between UA levels and Birth weight throughout pregnancy. **B** The relationship between UA and LBW risk of occurrence. **C** The relationship between UA and SGA risk of occurrence. Data were expressed as estimated OR values, 95% CIs (shaded areas), adjusted variables: age, education, family income, ART, smoking, BMI, parity, SBP, DBP, CHOL, TG, GH, PE, Anaemia, GDM, delivery method, DGW

By using the restriction cube spline model to analyze the relationship between UA levels and birth weight, birth weight decreased inversely with the risk of UA levels, especially around UA = 250 μmol/L, birth weight decreased faster (Fig. 2A), which suggested that high uric acid would limit fetal growth, so we explored the risk of LBW/SGA. As expected, UA levels were positively correlated with the risk of LBW/SGA, and significant cut-off point was also around UA = 250 μmol/L (Fig. 2B, C).

### Relationship between UA levels and LBW/SGA in different trimesters

**Fig. 3:**
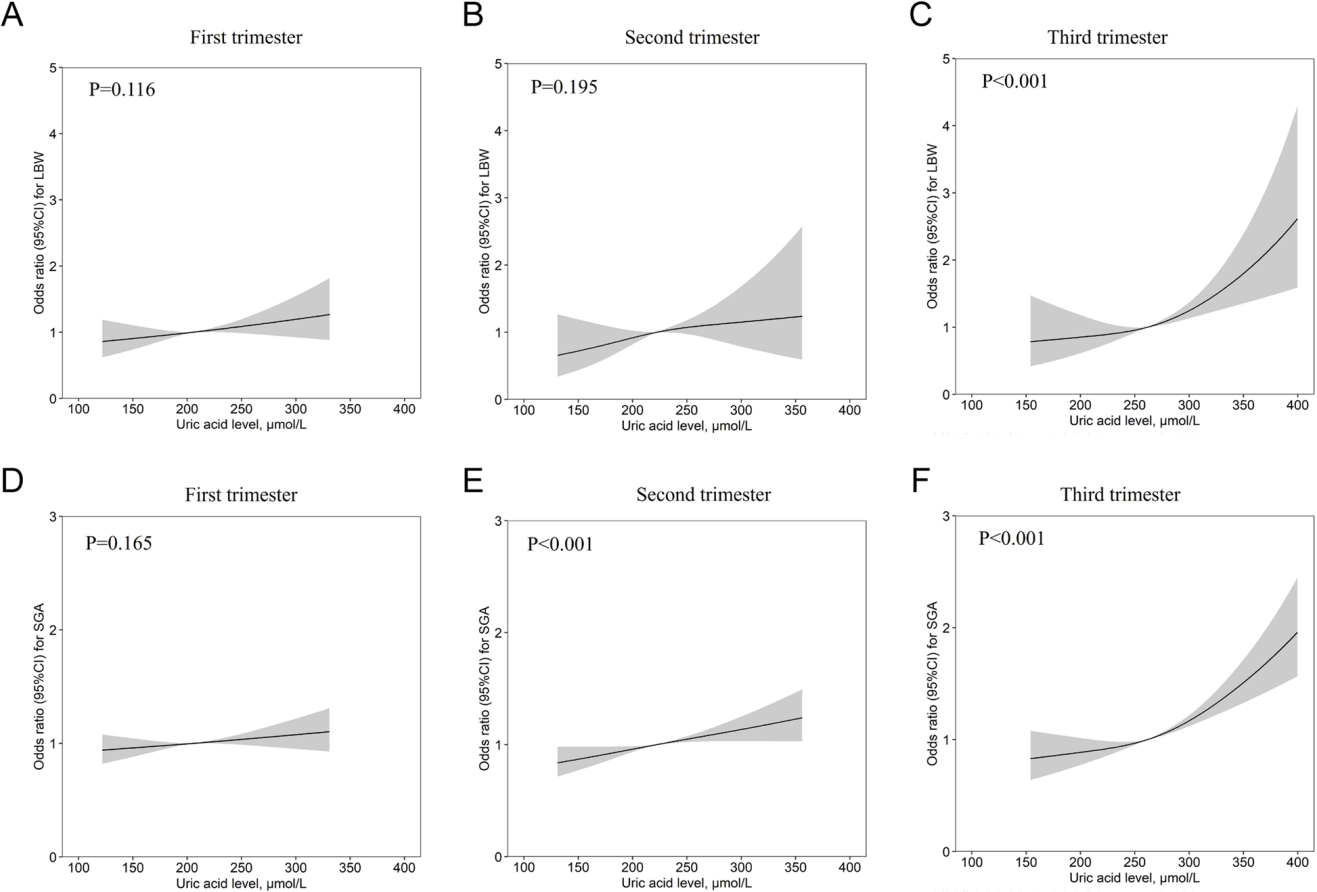
Association of maternal serum UA levels with Birth Weight throughout pregnancy. Association between UA levels and LBW/SGA risk in the first trimester (**A**/**D**), second trimester (**B**/**E**), and third trimester (**C**/**F**). Data were presented as estimated mean risk at 95%CI (shadowed area) and adjusted variables: age, education, family income, ART, smoking, BMI, parity, SBP, DBP, CHOL, TG, GH, PE, Anaemia, GDM, delivery method, DGW

Finding the right trimester to observe UA levels were essential to reduce the risk of LBW/SGA at birth. Therefore, UA levels in the first, second, and third trimester of pregnancy were studied. There was no statistical association between UA levels and the risk of LBW/SGA during the first trimester (Fig. 3A, D). In the second trimester, UA levels were positively associated with the risk of SGA (Fig. 3B, E). In the third trimester, the relationship between UA levels and the risk of LBW and SGA was both pronounced (Fig. 3C, F). Then the relationship between UA levels variation and the risk of LBW/SGA were examined in Tables 2 and 3. In Third-First, the risk of LBW increased significantly from the lowest quartile Q1 (UA variation** < **8.92 μmol/L) to the highest quartile Q4 (UA variation** ≥ **30.5 μmol/L) (*P*** < **0.001). The risk of LBW increased to 1.87-fold (aOR = 1.87, 95% CI, 1.57-2.21,* P*** < **0.001). The risk of SGA increased to 1.45-fold (aOR** = **1.45, 95% CI, 1.34, 1.56, *P*** < **0.001). In Second-First, from Q1 (UA variation < -34.7 μmol/L) to Q4 (UA variation ≥ -3.25 μmol/L), the risk of LBW was decreased to 0.55-fold (aOR = 0.55, 95% CI, 0.45, 0.78, *P* < 0.001). The risk of SGA was decreased to 0.71-fold (aOR = 0.71, 95% CI, 0.65, 0.78, *P* < 0.001). In Third-Second, a significant increase in the risk of LBW from the lowest quartile Q1 (UA variation** < **15.5 μmol/L) to the highest quartile Q4 (UA variation ≥ 66.0 μmol/L) was also discovered in Third-Second (*P*** < **0.001). The risk of LBW was increased to 1.95-fold (aOR** = **1.95, 95% CI, 1.59, 2.39, *P*** < **0.001). The risk of SGA was increased to 1.56-fold (aOR** = **1.56, 95% CI, 1.42, 1.71, *P*** < **0.001).

**Table 2:** Logistic regression analysis of UA variation and LBW

UA variation, μmol/L	Total(*n*=69,674)	LBW (*n* (%))	Crude model		Adjusted model	
			OR (95% CI)	*P*-value	OR (95% CI)	*P*-value
Third-First						
Q1(<8.92)	17,060	627(3.7)	ref		ref	
Q2(8.92-19.0)	17,017	379(2.2)	0.60(0.52,0.68)	<0.001	1.05(0.87,1.26)	0.614
Q3(19.0-30.5)	16,898	411(2.4)	0.65(0.58,0.74)	<0.001	1.30(1.08,1.56)	0.005
Q4(≥30.5)	17,294	727(4.2)	1.15(1.03,1.28)	0.012	1.87(1.57,2.21)	<0.001
missing	1405					
*P* for trend				0.002		<0.001
Second-First						
Q1(<-34.7)	11,000	474(4.3)	ref		ref	
Q2(-34.7-18.3)	11,022	298(2.7)	0.62(0.53,0.72)	<0.001	0.68(0.55,0.83)	<0.001
Q3(-18.3-3.25)	10,887	308(2.8)	0.65(0.56,0.75)	<0.001	0.56(0.45,0.69)	<0.001
Q4(≥-3.25)	11,136	458(4.1)	0.95(0.84,1.09)	0.467	0.55(0.45,0.68)	<0.001
missing	25,629					
*P* for trend				0.582		<0.001
Third-Second						
Q1(<15.5)	10,930	443(4.1)	ref		ref	
Q2(15.5-39.0)	10,871	264(2.4)	0.59(0.50,0.69)	<0.001	0.98(0.78,1.24)	0.893
Q3(39.0-66.0)	11,070	279(2.5)	0.61(0.53,0.71)	<0.001	1.30(1.04,1.62)	0.021
Q4(≥66.0)	11,126	513(4.6)	1.14(1.00,1.30)	0.042	1.95(1.59,2.39)	<0.001
missing	25,677					
*P* for trend				0.02		<0.001

Adjusted for variables: age, education, family income, ART, smoking, BMI, parity, SBP, DBP, CHOL, TG, GH, PE, Anaemia, GDM, delivery method, DGW

Third-First: UA levels variation between the third and first trimester. Second-First: UA levels variation between the second and first trimester. Third-Second: UA levels variation between the third and second trimester

**Table 3:** Logistic regression analysis of UA variation and SGA

UA variation, μmol/L	Total(*n*=69,674)	SGA (*n* (%))	Crude model		Adjusted model	
			OR (95% CI)	*P*-value	OR (95% CI)	*P*-value
Third-First						
Q1(<8.92)	17,060	1333(7.8)	ref		ref	
Q2(8.92-19.0)	17,017	1377(8.1)	1.04(0.96,1.12)	0.342	1.10(1.01,1.19)	0.023
Q3(19.0-30.5)	16,898	1462(8.7)	1.12(1.03,1.21)	0.005	1.16(1.07,1.26)	<0.001
Q4(≥30.5)	17,294	1965(11.4)	1.51(1.41,1.63)	<0.001	1.45(1.34,1.56)	<0.001
missing	1405					
*P* for trend				<0.001		0.001
Second-First						
Q1(<-34.7)	11,000	1271(11.6)	ref		ref	
Q2(-34.7-18.3)	11,022	976(8.9)	0.74(0.68,0.81)	<0.001	0.78(0.72,0.86)	<0.001
Q3(-18.3-3.25)	10,887	903(8.3)	0.69(0.63,0.76)	<0.001	0.73(0.67,0.81)	<0.001
Q4(≥-3.25)	11,136	918(8.2)	0.69(0.63,0.75)	<0.001	0.71(0.65,0.78)	<0.001
missing	25,629					
*P* for trend				<0.001		<0.001
Third-Second						
Q1(<15.5)	10,930	838(7.7)	ref		ref	
Q2(15.5-39.0)	10,871	943(8.7)	1.14(1.04,1.26)	0.007	1.18(1.07,1.30)	0.001
Q3(39.0-66.0)	11,070	941(8.5)	1.12(1.02,1.23)	0.023	1.14(1.03,1.26)	0.009
Q4(≥66.0)	11,126	1340(12.0)	1.65(1.51,1.81)	<0.001	1.56(1.42,1.71)	<0.001
missing	25,677					
*P* for trend				<0.001		<0.001

Adjusted for variables: age, education, family income, ART, smoking, BMI, parity, SBP, DBP, CHOL, TG, GH, PE, Anaemia, GDM, delivery method, DGW

Third-First: UA levels variation between the third and first trimester. Second-First: UA levels variation between the second and first trimester. Third-Second: UA levels variation between the third and second trimester

### Association between UA levels and the risk of LBW/SGA

Then, we wanted to determine whether different UA levels were associated with the risk of LBW/ SGA. In the quartile of UA levels, the risk of LBW increased significantly from the lowest quartile Q1 (UA** < **213.8 μmol/L) to the highest quartile Q4 (UA** ≥ **273.5 μmol/L) (*P*** = **0.001). This risk was significant in the third trimester of pregnancy (aOR = 1.79, 95%CI, 1.50, 2.14, *P*** < **0.001). The results were shown in Table 4. The risk of developing SGA also increased significantly from Q1 (UA** < **213.8 μmol/L) to Q4 (UA** ≥ **273.5 μmol/L) (*P*** < **0.001). In quartile Q4 (UA** ≥ **273.5 μmol/L), the risk also existed in the second(*P*** = **0.002) and third trimester (*P*** < **0.001) (Table 5).

**Table 4:** Logistic regression analysis of UA and LBW

UA, μmol/L	Total(*n*=69,674)	LBW (*n* (%))	Crude model		Adjusted model	
			OR (95% CI)	*P*-value	OR (95% CI)	*P*-value
Total						
Q1(<213.8)	17,391	504(2.9)	ref		ref	
Q2(213.8-242.0)	17,362	477(2.7)	0.95(0.83,1.07)	0.397	1.10(0.91,1.33)	0.315
Q3(242.0-273.5)	17,467	524(3.0)	1.04(0.92,1.17)	0.574	1.33(1.11,1.60)	0.002
Q4(≥273.5)	17,454	778(4.5)	1.56(1.39,1.75)	<0.001	1.57(1.31,1.87)	<0.001
*P* for trend				<0.001		0.001
First trimester						
Q1(<180.0)	14,588	374(2.6)	ref		ref	
Q2(180.0-205.0)	14,435	419(2.9)	1.14(0.99,1.31)	0.077	1.10(0.90,1.34)	0.355
Q3(205.0-234.0)	14,868	484(3.3)	1.28(1.12,1.47)	<0.001	1.18(0.97,1.43)	0.096
Q4(≥234.0)	14,733	551(3.7)	1.48(1.29,1.69)	<0.001	1.02(0.83,1.24)	0.871
*P* for trend				<0.001		0.76
Second trimester						
Q1(<194.0)	10,823	320(3.0)	ref		ref	
Q2(194.0-222.0)	11,033	344(3.1)	1.06(0.90,1.23)	0.487	0.95(0.77,1.18)	0.655
Q3(222.0-253.0)	11,105	388(3.5)	1.19(1.02,1.38)	0.025	1.07(0.86,1.32)	0.545
Q4(≥253.0)	11,084	486(4.4)	1.51(1.30,1.74)	<0.001	1.02(0.83,1.27)	0.837
*P* for trend				<0.001		0.589
Third trimester						
Q1(<227.5)	17,064	502(2.9)	ref		ref	
Q2(227.5-261.3)	17,055	433(2.5)	0.86(0.75,0.98)	0.023	1.17(0.97,1.42)	0.095
Q3(261.3-299.0)	17,081	464(2.7)	0.92(0.81,1.05)	0.209	1.34(1.11,1.61)	0.002
Q4(≥299.0)	17,069	745(4.4)	1.51(1.34,1.69)	<0.001	1.79(1.50,2.14)	<0.001
*P* for trend				<0.001		<0.001

Adjusted for variables: age, education, family income, ART, smoking, BMI, parity, SBP, DBP, CHOL, TG, GH, PE, Anaemia, GDM, delivery method, DGW

**Table 5:** Logistic regression analysis of UA and SGA

UA, μmol/L	Total (*n*=69,674)	SGA (*n* (%))	Crude model		Adjusted model	
			OR (95% CI)	*P*-value	OR (95% CI)	*P*-value
Total						
Q1(<213.8)	17,391	1352(7.8)	ref		ref	
Q2(213.8-242.0)	17,362	1466(8.4)	1.09(1.01,1.18)	0.022	1.09(1.01,1.18)	0.03
Q3(242.0-273.5)	17,567	1572(9.0)	1.17(1.09,1.27)	<0.001	1.18(1.09,1.28)	<0.001
Q4(≥273.5)	17,454	1893(10.8)	1.44(1.34,1.55)	<0.001	1.43(1.32,1.54)	<0.001
*P* for trend				<0.001		<0.001
First trimester						
Q1(<180.0)	14,588	1270(8.7)	ref		ref	
Q2(180.0-205.0)	14,435	1338(9.3)	1.07(0.99,1.16)	0.093	1.07(0.98,1.16)	0.12
Q3(205.0-234.0)	14,868	1328(8.9)	1.03(0.95,1.11)	0.494	1.03(0.94,1.11)	0.551
Q4(≥234.0)	14,733	1282(8.7)	1.00(0.92,1.08)	0.99	1.01(0.93,1.10)	0.816
*P* for trend				0.740		0.950
Second trimester						
Q1(<194.0)	10,823	951(8.8)	ref		ref	
Q2(194.0-222.0)	11,033	993(9.0)	1.03(0.94,1.13)	0.579	1.03(0.94,1.13)	0.542
Q3(222.0-253.0)	11,105	1072(9.7)	1.11(1.01,1.22)	0.027	1.14(1.04,1.25)	0.006
Q4(≥253.0)	11,084	1052(9.5)	1.09(0.99,1.19)	0.071	1.13(1.03,1.24)	0.013
*P* for trend				0.025		0.002
Third trimester						
Q1(<227.5)	17,064	1327(7.8)	ref		ref	
Q2(227.5-261.3)	17,055	1401(8.2)	1.06(0.98,1.15)	0.136	1.08(0.99,1.17)	0.078
Q3(261.3-299.0)	17,081	1466(8.6)	1.11(1.03,1.20)	0.007	1.13(1.04,1.23)	0.002
Q4(≥299.0)	17,069	1943(11.4)	1.52(1.42,1.64)	<0.001	1.49(1.38,1.61)	<0.001
*P* for trend				<0.001		<0.001

Adjusted for variables: age, education, family income, ART, smoking, BMI, parity, SBP, DBP, CHOL, TG, GH, PE, Anaemia, GDM, delivery method, DGW

### Synergy analysis

A stratified analysis of pregnant were performed to explore the influence of other factors on the relationship. First, we stratified the association of UA levels with the risk of LBW/SGA based on the BMI in the cohort. We found that in BMI** < **25 group, the incidence of LBW, SGA increased to 2.35-fold (95%CI, 1.66–3.31), 1.66-fold (95%CI, 1.37–2.00), respectively. In addition, for GH with hyperuricemia (UA** > **360 μmol/L) during pregnancy, the risk of LBW was 4.00-fold (95% CI, 2.01–7.93) higher than the control group and 2.63-fold (95%CI, 1.83–3.78) higher in SGA. Then, in the PE subgroup, we found significant additive interactions between PE and hyperuricemia (UA** > **360 μmol/L). When pregnant women had PE, the risk of LBW increased to 1.38-fold (95%CI, 0.63, 3.03) in individuals with hyperuricemia (UA** > **360 μmol/L). The risk of SGA increased to 1.81-fold (95%CI, 1.18–2.78). Furthermore, DGW also played an important role. Pregnant women were been grouped according to DGW. As compared to the control group, the hyperuricemic group (UA > 360 μmol/L) had 2.46-fold (95%CI, 1.62, 3.73) risk of LBW and 1.52-fold (95%CI, 1.24, 1.87) risk of SGA. For people with DGW < 37, the occurrence of high UA increased the risk of LBW to 2.70-fold (95%CI, 1.92, 3.80), while the risk of SGA increased to 2.13-fold(95%CI, 1.50, 3.02). The results were shown in Tables 6 and 7.

**Table 6:** Synergistic effects (LBW)

Variables	UA, μmol/L	Total (*n*=69,674)	LBW (*n* (%))	Crude model		Adjusted model	
				OR (95% CI)	*P*-value	OR (95% CI)	*P*-value
BMI, kg/m^2^							
No	<360	59,985	1768(2.9)	ref		ref	
	≥360	841	126(15.0)	5.80(4.77,7.05)	<0.001	2.35(1.66,3.31)	<0.001
Yes	<360	6428	225(3.5)	1.19(1.04,1.38)	0.014	0.54(0.43,0.68)	<0.001
	≥360	297	44(14.8)	5.73(4.14,7.91)	<0.001	1.10(0.60,2.02)	0.762
missing		22,855					
*P* for trend					<0.001		<0.001
GH							
No	<360	38,179	944(2.5)	ref		ref	
	≥360	455	29(6.4)	2.69(1.83,3.93)	<0.001	2.54(1.40,4.60)	0.002
Yes	<360	7914	342(4.3)	1.78(1.57,2.02)	<0.001	2.26(1.55,3.31)	<0.001
	≥360	271	73(26.9)	14.54(11.03,19.17)	<0.001	4.00(2.01,7.93)	<0.001
missing		22,855					
*P* for trend					<0.001		<0.001
PE							
No	<360	40,999	1068(2.6)	ref		ref	
	≥360	577	44(7.6)	3.09(2.26,4.22)	<0.001	2.10(1.25,3.53)	0.005
Yes	<360	2328	167(7.2)	2.89(2.44,3.42)	<0.001	1.06(0.69,1.63)	0.775
	≥360	144	58(40.3)	25.22(17.97,35.37)	<0.001	1.38(0.63,3.03)	0.418
missing		25,626					
*P* for trend					<0.001		0.226
DGW, weeks							
≥37	<360	64,865	629(1.0)	ref		ref	
	≥360	1013	30(3.0)	3.12(2.15,4.52)	<0.001	2.46(1.62,3.73)	<0.001
<37	<360	3584	1473(41.1)	ref		ref	
	≥360	212	151(71.2)	3.55(2.62,4.81)	<0.001	2.70(1.92,3.80)	<0.001

Adjusted for variables: age, education, family income, ART, smoking, BMI, parity, SBP, DBP, CHOL, TG, GH, PE, Anaemia, GDM, delivery method, DGW.

**Table 7:** Synergies effects (SGA)

Variables	UA, μmol/L	Total (*n*=69,674)	SGA (*n* (%))	Crude model		Adjusted model	
				OR (95% CI)	*P*-value	OR (95% CI)	*P*-value
BMI, kg/m^2^							
<25	<360	59,985	5532(9.2)	ref		ref	
	≥360	841	154(18.3)	2.21(1.85,2.63)	<0.001	1.66(1.37,2.00)	<0.001
≥25	<360	6428	345(5.4)	0.56(0.50,0.62)	<0.001	0.51(0.46,0.58)	<0.001
	≥360	297	38(12.8)	1.44(1.03,2.03)	0.035	0.90(0.62,1.30)	0.570
missing		2123					
*P* for trend					<0.001		<0.001
GH							
No	<360	38,179	3079(8.1)	ref		ref	
	≥360	455	57(12.5)	1.63(1.23,2.16)	0.001	1.70(1.27,2.28)	<0.001
Yes	<360	7914	856(10.8)	1.38(1.28,1.50)	<0.001	1.52(1.27,1.81)	<0.001
	≥360	271	67(24.7)	3.74(2.83,4.95)	<0.001	2.63(1.83,3.78)	<0.001
missing		22,855					
*P* for trend					<0.001		<0.001
PE							
No	<360	40,999	3383(8.3)	ref		ref	
	≥360	577	78(13.5)	1.74(1.37,2.21)	<0.001	1.69(1.30,2.19)	<0.001
Yes	<360	2328	305(13.1)	1.68(1.48,1.90)	<0.001	1.11(0.90,1.37)	0.328
	≥360	144	45(31.2)	5.05(3.55,7.20)	<0.001	1.81(1.18,2.78)	0.007
missing		25,626					
*P* for trend					<0.001		0.006
DGW, weeks							
≥37	<360	64,865	5559(8.6)	ref		ref	
	≥360	1013	124(12.2)	1.49(1.23,1.80)	<0.001	1.52(1.24,1.87)	<0.001
<37	<360	3584	517(14.4)	ref		ref	
	≥360	212	83(39.2)	3.82(2.85,5.11)	<0.001	2.13(1.50,3.02)	<0.001

Adjusted for variables: age, education, family income, ART, smoking, BMI, parity, SBP, DBP, CHOL, TG, GH, PE, Anaemia, GDM, delivery method, DGW

## Discussions

Birth weight is not only an important indicator of the fetus's growth and nutritional status, but also an important determinant of its growth, development, and health status after birth. In previous studies, the relationship between UA levels and birth weight was controversial [15–18]. In this large, population-based retrospective cohort study of more than 60,000 Chinese women, UA was found negatively associated with birth weight. High UA levels were an independent risk factor for the risk of LBW/SGA. And the higher the maternal UA levels, the higher the risk of LBW/SGA.

UA, the final product of purine degradation, has excellent antioxidant activity at physiological concentrations. It is important to note, however, that UA is a proinflammatory factor and causes endothelial dysfunction, which adversely affects the placenta during pregnancy. [21, 22] There were some evidence that elevated plasma UA could cause stillbirth, neonatal death, preterm birth, LBW, SGA, etc. [23] It is common for pregnant women who have high UA levels during pregnancy to suffer from diabetes, hypertension, and preeclampsia. [12, 24] Multiple studies demonstrated that high UA levels could predict birth weight in patients with gestational hypertension and preeclampsia. [25, 26] The possible mechanisms could as followed: (1) During pregnancy, high levels of UA inhibit amino acid transport, which resulted in fetal growth restriction; [27] (2) UA could activate inflammasome in the trophoblastic layer, produce IL-1β, induce inflammation at the maternal-fetal interface, and lead to placental dysfunction and adverse pregnancy outcomes; [7] (3) UA could deposit the placenta, aggravate fetal placental circulatory disorders, and indirectly or directly affect the fetus; (4) Higher or lower UA levels impaired fetal growth and oxidative stress balance. However, most previous studies focused only on the effect of hyperuricemia on birth weight in pregnant women in the setting of hypertension, and the relationship between hyperuricemia in normal blood pressure and birth weight has rarely been elucidated. This study still found a positive correlation between UA levels and the risk of LBW/SGA after adjusting for factors such as blood pressure, and the large population base of the study further demonstrated the accurate confidence of the conclusions. At the same time, we confirmed that pregnant women with GH or PE complicated by high UA levels were at significantly increased risk of developing LBW/SGA. In addition, the effect of high UA levels on the risk of LBW/SGA was also significant whether in DGW** < **37 or DGW** ≥ **37. And this correlation was also present in BMI** < **25 group. This may be related to maternal nutritional status.

In our study, we looked more closely at the relationship between UA levels and the risk of LBW/SGA during different trimesters which outperformed previous studies that only focused on maternal UA levels in one trimester [27, 28]. By analyzing UA levels throughout pregnancy and adjusting for confounding factors, we found that high UA levels were still associated with the risk of LBW/SGA. Our findings suggested that UA levels were also associated with the risk of SGA in the second trimester, and in the third trimester, UA levels were associated with the risk of LBW/SGA. Additionally, UA levels variation was associated with the risk of LBW/SGA. Therefore, we believed that more attention should be paid to UA levels especially the variation during pregnancy in order to reduce the incidence of LBW/SGA earlier and more effectively.

Nevertheless, there were some limitations to this study. First, it was retrospective, thereby limiting causality. Further, this study only included Chinese of ethnic homogeneity dominated by Han Chinese, making it difficult to generalize. Therefore, we suggested that future well-designed large, multicenter prospective longitudinal studies should be conducted to accurately identify the causal relationship between UA levels and birth weight as early as possible, and to identify and prevent adverse fetal outcomes in pregnant women with hyperuricemia as soon as possible. To better understand how UA levels affect pregnancy and fetus, future studies should examine serum UA levels before and after pregnancy. In addition, it is also necessary to assess the impact of the level of the UA levels variation on the risk of LBW and SGA.

## Conclusions

This cohort study demonstrated a noteworthy positive association between uric acid levels and the risk of LBW and SGA. Moreover, pregnant women with GH, PE, and BMI** < **25, complicated by hyperuricemia, exhibited a significantly high risk of LBW/SGA. And whether it is DGW < 37 or DGW ≥ 37, if UA** > **360 μmol/L, the risk of LBW/SGA would increase. These findings hold considerable significance for the provision of pregnancy care. We further underscored the necessity for robust longitudinal studies to elucidate underlying mechanisms and ascertained the optimal timing for interventions, particularly among diverse ethnic groups and populations.

## Data Availability

The datasets generated and/or analyzed during the current study are not publicly available as the additional results from the study are yet to be published. However, they are available from the corresponding author on reasonable request.

## Abbreviations

UA: Uric acid
LBW: Low birth weight
SGA: Small for gestational age
ART: Assisted reproductive technology;
BMI: Body mass index
SBP: Systolic blood pressure
DBP: Diastolic blood pressure
CHOL: Cholesterol
TG: Triglyceride
GH: Gestational Hypertension
PE: Preeclampsia
GDM: Gestational diabetes mellitus
AGA: Appropriate for gestational age
LGA: Large for gestational age
